# Pathways into homelessness and perspectives on prevention: A qualitative study of army veterans

**DOI:** 10.1371/journal.pone.0342060

**Published:** 2026-03-02

**Authors:** Katherine A. Koh, Jenny D’Olympia, Dina Hooshyar, Ana-Maria Vranceanu, Sara Garde, Zachary Ginsburg, Kelly Ré, Michaela Spaulding, James A. Naifeh, Matthew K. Nock, James Wagner, Robert J. Ursano, Jack Tsai

**Affiliations:** 1 Department of Psychiatry, Massachusetts General Hospital, Boston, Massachusetts, United States of America; 2 Boston Health Care for the Homeless Program, Boston, Massachusetts, United States of America; 3 William James College, Newton, Massachusetts, United States of America,; 4 United States Department of Veterans Affairs National Center on Homelessness among Veterans, Washington, District of Columbia, United States of America; 5 Department of Psychiatry, University of Texas Southwestern Medical Center, Dallas, Texas, United States of America; 6 Center for Health Outcomes and Interdisciplinary Research, Department of Psychiatry, Massachusetts General Hospital, Boston, Massachusetts, United States of America; 7 Center for the Study of Traumatic Stress, Department of Psychiatry, Uniformed Services University of the Health Sciences, Bethesda, Maryland, United States of America; 8 Henry M. Jackson Foundation for the Advancement of Military Medicine, Inc., Bethesda, Maryland, United States of America; 9 Department of Psychology, Harvard University, Cambridge, Massachusetts, United States of America; 10 Survey Research Center, Institute for Social Research, University of Michigan, Ann Arbor, Michigan, United States of America; 11 United States Department of Veterans Affairs, National Center on Homelessness among Veterans, Washington, District of Columbia, United States of America; 12 School of Public Health, University of Texas Health Science Center at Houston, Houston, Texas, United States of America; 13 Department of Psychiatry, Yale University School of Medicine, New Haven, Connecticut, United States of America; University of Missouri School of Medicine, UNITED STATES OF AMERICA

## Abstract

**Background:**

Despite a national focus on ending veteran homelessness, many veterans remain homeless in the United States. Little qualitative research has explored in national samples the pathways that lead to homelessness among veterans or veteran perspectives on homelessness prevention.

**Methods:**

We conducted individual semi-structured interviews with 55 US Army veterans from the national Army Study to Assess Risk and Resilience in Servicemembers-Longitudinal Study (STARRS-LS) who reported having an episode of homelessness after separating from the Army. Interviews included questions about their pathways into homelessness and what could have prevented their homelessness. Interview transcripts were analyzed for themes using a hybrid deductive-inductive approach.

**Results:**

We identified three main themes as pathways into homelessness: (1) *financial challenges while reintegrating into civilian life*, including the subthemes of difficulty finding adequate employment and lengthy processing of VA disability benefits; (2) *planning challenges around the transition,* including the subthemes of lack of awareness of existing resources and personal challenges with planning; and (3) *interpersonal disruptions upon reintegration*, including the subthemes of loss of a primary relationship and intersections between social disruptions and having a mental illness. Notably, many participants identified connections between multiple themes as leading to homelessness after service. Veterans expressed that enrolling veterans in services prior to separation, decreasing barriers to receiving VA disability benefits, and enhancing access to mental health care would have helped prevent their homelessness.

**Conclusion:**

The multiple challenges veterans identified as leading to homelessness most often began during the immediate transition period from the US Army to civilian life and are modifiable. These findings can be used to develop or enhance systems to improve prevention of homelessness among veterans.

## Introduction

Homelessness among veterans is a major problem in the United States. A public area of interest for over two decades, ending veteran homelessness remains a focus of many government initiatives [1,2]. While the Department of Veteran Affairs (VA) and other federal and community partners across the country have made substantial progress in reducing veteran homelessness by 56% since 2010 [3], sizable numbers of veterans remain homeless. As of January 2024, 32,882 US veterans were without homes on any given night [3].

Research has indicated that veterans are at greater risk of homelessness than other adults. Despite being older, better educated, more likely to have married, and more likely to have health care coverage than other homeless adults, risk of homelessness remains elevated among veterans [4]. Mental illness and substance use disorders have been identified as the strongest and most consistent risk factors for veteran homelessness, followed by low income and other income-related factors [5]. However, the exact pathways or processes explaining *how* such factors may lead to veterans becoming homeless, and the role that post-military factors play in this process, remain unclear [5]. The vast majority of studies on this topic have relied on descriptive quantitative data, with a paucity of qualitative research, except for a few notable studies. For instance, one qualitative study of post 9/11-era veterans found that veterans mainly see their homelessness as rooted in non-military, situational factors, such as unemployment and the loss of relationships [6]. Another study of female homeless veterans, whose service preceded 2001, suggested that homelessness resulted from a combination of pre-military (childhood adversity), military (trauma and substance use), post-military (adversity, mental health, and medical problems), and unemployment factors [7].

In addition, there has been a notable dearth of qualitative studies focusing on homelessness *prevention* in veterans—i.e., asking veterans directly their perspectives on what could have been done to prevent their homelessness. Qualitative research on not only pathways to homelessness, but also what the Department of Defense (DoD) or VA could do to help prevent veteran homelessness is a valuable opportunity to inform program development. For instance, since 1991, the military has implemented the Transition Assistance Program (TAP), run jointly by the DoD, Department of Labor, and VA. TAP is designed to help military service members transition to civilian life through a structured program consisting of classes and workshops focused on employment, education, and post-military goals [8]. Though promising, the TAP program has undergone various evolutions and at times has been criticized for lack of personalization, inconsistent quality, and unclear outcomes [8]. Research of this type could provide unique information about how the TAP and other programs can help alter risk of homelessness among transitioning veterans. Moreover, existing research on veteran homelessness has mainly been limited to recruitment of veterans engaged with the VA healthcare system; however, nearly 40% of veterans do not use VA healthcare services, demonstrating the need to expand research beyond VA patients [9].

We addressed these important knowledge gaps by carrying out a descriptive qualitative study [10] of the pathways leading to homelessness and veteran perspectives on what could have helped prevent their homelessness.

## Methods

### Recruitment

We recruited our sample from the Army Study to Assess Risk and Resilience in Soldiers-Longitudinal Study (STARRS-LS), a national and prospective study of Army soldiers [11]. STARRS-LS overall included a cohort of nearly 9,000 separated post 9/11 soldiers who completed surveys both shortly before military separation and then again after separation. Participants in STARRS-LS were initially surveyed while on active duty between 2011–2013 and then re-surveyed at multiple subsequent time points. These participants gave written informed consent at the time of recruitment to be recontacted for future research.

In this study, we added a qualitative component to STARRS-LS by interviewing participants who reported having an episode of homelessness after separating from military service. The third wave of surveys, STARRS-Longitudinal Study 3 (STARRS-LS3), was collected during the years of 2020–2022. Participants who answered “yes” to whether they had been homeless since separating from the Army in this survey were eligible to participate in the current study. These participants therefore separated from the Army anywhere between 2011–2022. Of note, most of these individuals were no longer homeless at the time of interview.

We sent an email to these participants on September 25, 2023 and recruited until January 22, 2024, informing them that we were interested in learning more about individuals who experienced homelessness post-separation to help prevent homelessness in other soldiers at risk for homelessness. All participants provided written informed consent. All recruitment and interview procedures were approved by the University of Michigan (U-M) IRB. U-M is part of the multi-institutional team of researchers that designed and conducted the STARRS study, along with Harvard Medical School and the Uniformed Services University. We adhere to the COREQ guidelines [12] in describing our methods, results, and discussions.

### Sample

Email invitations were sent to 597 veterans, of which 119 (19.9%) completed an interest form. Participants who filled out an interest form were contacted to explain the study, obtain informed consent, and schedule a recorded interview. Participants were told that the interviewer would be from the STARRS-LS study team and would only know their first name and no other identifying information. Furthermore, participants were informed that the study team would remove any identifying information from the transcript and that all de-identified transcripts would then be analyzed to learn more about how to reduce veteran homelessness. Ninety-five interviews were scheduled, of which forty interviews (42%) were either cancelled or participants did not attend. If the participant did not attend their initial interview, the U-M scheduler reached out to re-schedule the appointment. Fifty-five interviews were completed between October 2023-February 2024.

### Development of interview guide

A semi-structured interview guide was created based on a review of both quantitative and qualitative studies examining factors that lead to veteran homelessness, as well as consultation with two veterans who were members of the study team. We specifically developed the script to understand not only what led to homelessness but what the Army or VA could have done to prevent it. The final interview script consisted of five questions that asked about the transition from military to civilian life including any unique challenges veterans faced, what led them to becoming homeless after separating from the Army, what their experience of homelessness was like, and what could have prevented them from becoming homeless (Table 1). The semi-structured format allowed for richness in response and for the interviewer to follow up with further questions as needed.

**Table 1:** Semi-Structured Interview Guide.

1. What was the process of transitioning from military to civilian life like for you? What were the challenges you faced?
2. What led you to becoming homeless after leaving the Army?
a. NOTE if people have multiple instances of being homeless after leaving the Army, focus on the *first* time since leaving the Army.
3. Please tell us about what your experience of homelessness was like after leaving the Army.
4. What do you think either the Army or VA could have done to help prevent you from becoming homeless in the first place?
5. Is there anything else you can think of that the Army or VA should do to prevent veterans from becoming homeless?

### Data collection

Interviews were performed virtually via Zoom and took 30–60 minutes. The interviewers were four graduate students in the military psychology track at William James College (co-authors SG, ZG, KR, MS). Two of these interviewers are veterans, and the other two had previous employment serving veterans. Interviewers were specifically trained in conducting and analyzing qualitative interviews by two supervisors—a psychiatrist with expertise in homelessness (lead author KK) and a psychologist who is an Air Force veteran with expertise in veteran mental health (second author JD). A program coordinator joined participants at the beginning of the interview to discuss logistics (e.g., recording, length of time, etc.). The participant and interviewer were subsequently alone during the interview. Each interview was audio recorded and transcribed using voice-recognition software. Participants were given the option to turn their camera off during the interview to maximize comfort. The team checked each transcription with the digital recording for accuracy, making edits as necessary. Transcripts were not returned to the participants for comment. No repeat interviews were conducted. All participants received $50 for their participation.

### Data analysis

Qualitative analysis was based on a hybrid deductive-inductive approach, a type of thematic analysis [13]. This included all supervisors and interviewers familiarizing themselves with the data and generating initial codes based on concepts from existing homeless veteran literature. We summarized the data and identified initial themes. We then added inductive coding, which emerged from participant interviews. We discussed researcher reflexivity using collaborative reflection within our study team, intentionally examining our own point of view and how this could impact outcomes of the research. For instance, as all members of the study team were mental health professionals, we discussed how we may be more likely to interpret ambivalent comments about a therapist or psychiatrist in a more positive light.

Transcripts were split up to be reviewed by the four interviewers, and 20% of the transcripts were checked for reliability by the two supervisors, who kept track of the percent agreement. The two supervisors then met regarding how they coded the text and resolved differences by discussion. De-identified/coded transcripts were initially stored at the U-M on secure processing servers and then transferred to a secure HMS STARRS-LS server. We used Microsoft Excel to organize the data by question. The supervisors met regularly to review codes and group them into themes and subthemes by first connecting similar codes into categories and then searching for broader patterns that emerged from the data. Themes and subthemes were linked to key quotes and then corroborated, refined, and finalized by the two supervisors.

## Results

Characteristics of the participants were obtained from STARRS LS3 survey responses and confirmed at the start of the interview (Table 2). In terms of demographics, 10.9% of participants were female, 12.7% were Hispanic, 21.8% were Non-Hispanic Black, and 58.2% were Non-Hispanic White. The mean duration of military service was 7.8 years. The mean length of time between separating from the Army and becoming homeless was just over one year and mean length of homelessness was 4.9 months. In terms of type of homelessness, 87.3% slept with friends or family, 49.1% slept in a motel or hotel, 36.5% slept in another place not intended for residence, and 16.4% slept at an institution (note more than one answer was possible).

**Table 2:** Demographic, Military, and Homelessness-Related Characteristics of Veteran Participants.

Age at time of LS3 survey	Mean = 38.2
Age at separating from Army	Mean = 29.5
Age at first homelessness after separating from Army	Mean = 30.8
Gender at LS3	Male: 85.5% Female: 10.9%
Race/ethnicity	Non-Hispanic White: 58.2% Non-Hispanic Black: 21.8% Hispanic: 12.7% Other: 7.3%
Education	Alternative certificate or less than HS: 3.6% HS or GED: 78.2% Some college: 3.6% College or more: 14.6%
Marital status	Currently married: 50.9% Never married: 41.8% Previously married: 7.3%
Duration of military service	Mean = 7.8
Rank at separation	Enlisted service member: 56.4% Non-commissioned officer: 41.8% Commissioned officer: 1.8%
Military separation status	Chose to leave: 40.0% Army did not extend service commitment/did not promote/not eligible or recommended for reenlistment: 5.45% Combination of both: 5.45% Other or unknown: 49.1%
Length of time between active duty service and first becoming homeless	1 = immediately after 2 = within 30 days 3 = within 1 year 4 = within 2 years 5 = more than 2 years later Mean = 3.1; Median = 3, Mode = 3 (within one year of separating)
Total months homeless during first episode since separating from Army	Mean = 4.9
Location slept when homeless	At friend’s/family: 87.3% Motel or hotel: 49.1% Another place not intended for residence: 36.5% In an institution: 16.4% *Note more than one answer possible
Number of times homeless since separating from Army	Mean = 1.7

**Fig 1:**
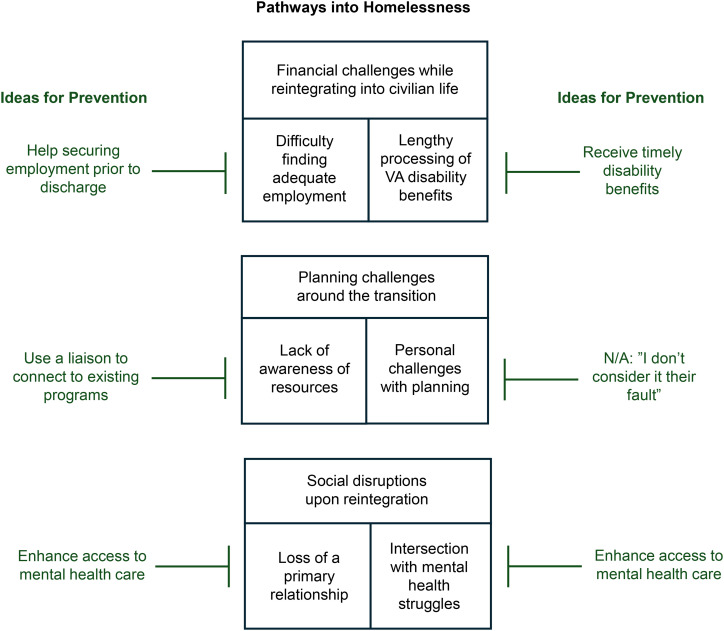
Pathways into Homelessness and Ideas for Prevention.

The qualitative analysis yielded three primary pathways to homelessness: (1) financial challenges while reintegrating into civilian life, (2) planning challenges around the transition, and (3) interpersonal disruptions upon returning to civilian life. These pathways, subthemes emerging within each, and veteran perspectives on prevention are described below and illustrated in the Fig 1.

### Pathways to homelessness

#### Theme 1) Financial challenges while reintegrating into civilian life.

Financial challenge while reintegrating into civilian life, i.e., not having enough money to cover housing, was the most common theme identified. The two subthemes in this area were difficulty finding adequate employment to provide sufficient income and the lengthy processing of VA service-connected disability benefits.

##### Subtheme 1A: Difficulty finding adequate employment:

Participants expressed difficulty in translating the skills they learned in the Army into civilian life, resulting in challenges finding and securing employment. This led them to be without work—and therefore income—during the critical early stages of transition. Several participants noted that a challenge of finding work was feeling unprepared due to a routine in the Army that abruptly ended when entering civilian life.

Participant 39: *“I’d say probably one of the hardest things transitioning out and finding work was a lot of it is just the culture shock. Because you’re going from a military environment and a way of life. Back to a civilian way of life and you just don’t fit in anymore. Nothing feels right. The people don’t feel right. The situation doesn’t ever feel right. Everything is uncomfortable.”*

Exacerbating the incongruence between the skills possessed and skills needed, joining the Army at a young age often came up, as many had not had any previous employment prior to entering the Army.

Participant 41 noted: *“I think, my general experience with transition was pretty difficult. I was [21–25 years old]. And I had saved up a lot of money from being in the Army. And I didn’t know how to spend it. I did not invest it. I never had a job before the Army. So a little hard for me to find work or to understand like the life balance and stuff…”*

Notably, even when employment was found, the job was often not enough to pay for the high cost of housing. Multiple participants also noted working multiple jobs.

Participant 53: *“… I ended up living out of my car for a while working a restaurant job. But I wasn’t making enough money to get an apartment or anything like that. Because the cost of living in that area was so high... and the hours were not good as well.”*

Most participants who were staying with family and friends did not describe finding work while homeless to be as strenuous as those in other homeless living situations. For instance, participant 23 noted of his homeless experience, “*I mean, I guess it wasn’t too bad… We stayed with friends kind of all over the country… And a friend of mine’s son was selling his house… So like my first job was basically like helping out with the work to fix the house up*… *Which I then eventually bought.*”

This was contrasted with participant 53 stated, who stayed in his car: *“The biggest struggle was I think staying presentable so to speak, because I was working after a bit… And so basically I was trying to like try and stay like looking like I wasn’t homeless for my job… So I showered like twice a week… But I was always like trying to like figure out you know like where I could park and not get… the cops called on me by people that live around there and stuff like that.”*

##### Subtheme 1B: Lengthy processing of VA disability benefits:

A second subtheme that emerged in this area was the significant challenges participants experienced navigating the process for applying for disability ratings, citing unclear guidance and contradictory information through the claims process. These barriers and misunderstandings resulted in an extended period in which they were counting on being eligible for benefits only to find out they were ineligible, leading to a confusing situation and additional financial difficulties.

Participant 2: *“I was denied my disability by the VA until [YEAR]. I tried to apply, Late [YEAR], early [YEAR] but I was told I didn’t qualify because my time of service was too short. But I guess whenever they looked that most of my time in the service was in [THEATER]. So then they were contradicting themselves and saying the time out of country counts as double or something like that, it weighs it different or something. I don’t know.”*

For those who qualified for VA service-connected disability post-separation, many participants still noted they became homeless in part due to a delay in receiving their benefits. After applying, the lag in being approved and receiving pay was substantial, lasting months to over a year in some cases. There was also a lack of clarity about when they should expect to receive the payment.

Participant 24: *“We also, as far as my benefits after I got out of the Army from the VA side, they never told me when those were going to start being received… so I was without pay for quite a few months and that affected me being able to take care of rent, bills, things like that. So that essentially was part of why I ended up becoming homeless...”*

Others suggested that the lengthy process and lack of clarity persisted even a decade later when submitting supplemental claims.

Participant 43: *“I didn’t have anybody saying they should do this or having a kind of recommendations of what I should list for injuries that I sustained while in service or anything like that. So it’s all on my own. Even right now. This is almost 10 years later. I’m still submitting supplemental claims to my original claim because for one instance, I had a surgery on my left hand while I was in a training exercise in [OCONUS LOCATION]. And they sent back on my original claim that that injury did not happen, was on active duty... I have an appointment to submit that claim… and it’s been [> 6 to 10 years]. So it has not been easy.”*

#### Theme 2) Planning challenges around the transition.

The next main theme identified as a pathway to becoming homeless after separating from the Army was planning challenges, including subthemes of lack of awareness of existing resources upon transitioning and personal challenges with planning.

##### Subtheme 2A: Lack of awareness of existing resources upon transitioning:

Participants noted that though they took transition classes prior to leaving active duty through the TAP, they felt the information they received was not adequate and the classes did not clearly describe the programs available to help soldiers after leaving active duty.

Participant 39: *“… I mean, they, what we did go through mandatory, it was sort of lackluster and really inadequate. The programs that the military has or had, when I got out are geared more toward your senior enlisted and your officers. We didn’t, I didn’t even know it existed until 2 years after I was already out of the military. Which to me is ridiculous and it’s still that way and I have a lot of buddies and across all branches that, you know, are my rank or equivalent or less and they are saying all the same thing. That you don’t even know these programs exist until 2 years after you get out a lot of times.”*

Other participants noted that though VA programs to support the transition existed, this was not clearly communicated to soldiers and it was not known how to access them. Various VA programs have outreach components for new veterans depending on their needs, but as Participant 1 reported: “*I know there’s a lot of VA programs out there and stuff, but they don’t really advertise them.”*

Some veterans had the impression that once they left the military, they were done with the military and did not think of seeking resources related to their military background when they were homeless.

Participant 24: *“I didn’t know what else there was out there as far as you know, like I said, if I needed a place to go, I didn’t know what benefits there were for that. I didn’t know that both the Army and the VA could help me you know, to find shelter. I didn’t know that, you know, there were education benefits until later on, which now I’m, I’m utilizing that.”*

The issue of age was again mentioned in this context. Several participants noted joining the Army right after high school and therefore not being prepared after coming out.

Participant 5: “… *When I joined the military I was 18 years old, like I had never been on my own ever at like 18… never, you know, lived on my own… From what I remember they talked about the loan stuff, but they didn’t really talk about the process of buying a house right so, or even getting into an apartment.*”

##### Subtheme 2B: Personal challenges with planning:

It is notable that some participants stated that they felt the inadequate planning was their fault, rather than that of the Army. Participants noted that they had unrealistic expectations and did not anticipate how hard it would be to find employment or manage money during reintegration. These findings were distinct from the lack of awareness listed above, as they revealed self-insights about why they may have struggled. For instance, one veteran shared that while he took classes, he had difficulty paying attention.

Participant 11: *“I want to say I’m going to take personal responsibility for not knowing how to manage my finances. I would think my common sense tells me [that they went over preparing a plan or applying to jobs], but I was not able to pay attention in class.”*

Another participant noted that he could have planned better and bought a house sooner, but this was difficult because he did not have a job and therefore did not know where he was going to live, demonstrating intersecting factors:

Participant 31: *“You know, I guess, it could be lack of planning, I guess, but it’s hard to, I think secure additional housing. I guess I could have done that. I guess we could have bought a house before we got out, but we didn’t know where we were gonna live because I didn’t have a job yet. So I think that’s part of [why] the job insecurity piece led to a delay in housing for us.”*

Many of these individuals were explicit in stating that it was not the Army’s fault and that there was nothing that could have been done to prevent their homelessness.

Participant 37: *“That’s a hard question because I’m big on self-accountability, so like, I, I don’t consider it their problem, you know, or that their fault, you know.”*

Relatedly, others stated that it was hard to accept help. The military culture’s focus on self-reliance may have influenced veterans in this respect.

Participant 4: *“My pride would not have allowed it. Like I said, when I got out. I really didn’t wanna have anything to do with government stuff for a while.”*

Notably, one participant mentioned that military culture influenced him to think he could do something that he should not actually do.

Participant 14: *“Just bad planning on my part. I decided it would be a good idea to try and do a vacation. You know, to a foreign country that I didn’t really have all the money for. So, definitely I guess is a bit of that still had that like machismo about, I’m in the infantry, you know, what’s to stop me? And then getting over there and being like, yeah, I don’t have the money I need. I don’t have you know the various supplies that I need. So that kind of dumb part essentially is what led to it. Nothing drug or alcohol related just being a real dumb one, that’s all.”*

#### Theme 3) Interpersonal disruptions upon returning to civilian life.

The final main theme identified as a pathway to becoming homeless after separating from the Army was interpersonal disruptions, including subthemes of loss of a primary relationship and an intersection between these social disruptions and mental health struggles.

##### Subtheme 3A: Loss of a primary relationship:

Multiple participants highlighted the profound impact of losing primary relationships during the transition. They described the financial and emotional difficulties of managing breakups, separations, divorce, and death as key factors contributing to their homelessness.

Participant 48: *“You know, my wife left. And took my kids to [STATE]... I ended up not getting any work and so a lot of time passed by. And right about at the same time the unemployment stopped is when I finally got someone to sell our house... So she had started the divorce out of state… Now this money that I was really banking on ends up going into a trust in the state of [STATE]. I’m like kind of behind on a court case that I didn’t really know existed. So I ended up getting a lawyer and that was like all the money I had. And so I, you know, just kind of ended up me being on the streets.”*

Several participants again noted that joining the military at a young age, not long after high school, made it particularly difficult to re-establish social relationships after separating from the military. Their trajectories had abruptly shifted due to military service and it was difficult to return to their prior life.

Participant 21: *“I guess mentally too just trying to figure out, you know, your life outside of the military, cause you join at a certain age at [<21], you know, your whole life changes. Then once you out, you know. Your life has changed, your friends is different, everything is different, you try to come back home and hang with them, but you can’t fit in because you don’t fit in no more. Just all that type of stuff.”*

As above however, participants also attributed several converging factors, for instance going through a divorce while simultaneously struggling to find adequate employment, further compounding the challenge of maintaining housing.

Participant 25: *“It was nowhere near enough time to have enough money… I didn’t have relationship anymore, I didn’t. So it was trying to come to terms with my family and be for a little while and then trying to find another job. Which wasn’t as easy as I thought to be. It was hard trying to transition into the workforce. Oh, that was challenging as well.”*

##### Subtheme 3B: Connection between mental health and relationship struggles:

Several participants identified a bidirectional relationship between mental health struggles, such as alcohol use disorder and bipolar disorder, and relationship loss, including death, as leading to homelessness. Some revealed that the mental health issues led to relationship loss.

Participant 21:*“Basically my mental health caused me to get a divorce, somewhat. You know I had to leave and I didn’t have, you know, we were staying together, but when I left, you know, I think I was like in a manic state, so I wasn’t really thinking straight. It hit me once you start realizing you gotta be in a hotel and then you got to think about funds…”*

Others indicated that the relationship loss led to mental health and substance use issues. For instance, participant 6 described a complex journey that included using drugs due to the death of his best friend from the Army just after getting out as a way to “*numb myself*.” He stated that it was normal to drink every day in the infantry and that he and his military buddies kept drinking together when they returned to civilian life, which evolved into hard drug use. He was working odd jobs, had a delay in receiving disability payment, and then six months later ended up getting a back payment. With this large sum of money he quit his job, moved into a girl’s place, and was kicked out due to drugs. He then moved in with his parents and was eventually kicked out of his parents’ house due to drug use. Though he was able to rent a room, he eventually ended up sleeping in a car due to his ongoing drug use.

### Perspectives on prevention

Veteran perspectives on prevention yielded three main themes: (1) enroll veterans in services prior to separation, (2) decrease barriers to receiving VA disability benefits, and (3) enhance access to mental health care.

#### Theme 1: Enroll veterans in services prior to separation.

Participants suggested that the Army could have helped prevent homelessness by helping connect them to sustainable employment and other programs prior to separation. Several people expressed the importance of having a job soon after leaving active duty service and that the Army could help facilitate that.

Participant 39: *“I would say probably the most important thing is the ability to get the foot in the door with a company that is going to be located, you know, where they want to go. You know, somehow some kind of link to get them established with a hiring team. You know, not saying that they should just be sliding vets right into positions. They should still be qualified they should still have to go through the interview process, but maybe starting that process while they’re still in service.”*

Participants noted that the transition classes should not just inform transitioning veterans about services, but actually enroll them in services before leaving.

Participant 39: *“There’s a lot of stuff that’s in there that you don’t know about they don’t go over it a lot of the stuff that you have available to you should be implemented before you get out. The schooling, the post 9 11 GI bill, the usage, how to use it all of that stuff I mean, it’s, they briefly go over it, but it’d be better if they actually got people enrolled and have them pick their school out.”*

Participants commented that the Army or VA could help prevent homelessness by providing a liaison to help them navigate existing services. Although most VA medical centers do have such liaisons, some participants appeared not to be aware of them. Another participant suggested that the liaison should start even before leaving active duty to be able to help connect to resources and leave only once the veteran feels settled.

Participant 10: *“Yeah, cause honestly it if they, if they have some type of portion where like right before you get out where you know, if someone you know, side by side understanding the situation and trying to make sure you’re good before letting go are, you know, at least a month. You know, just showing the resources and actually seeing how everything is going.”*

#### Theme 2: Decrease barriers to receiving VA disability benefits.

Participants noted that the most effective way to prevent homelessness would be to decrease barriers to receiving disability, including the VA recognizing eligible service-connected disabilities and having a speedier processing time for approval and receipt of VA disability benefits.

Participant 41: *“I think within the last year I got rated as a 100% permanently and totally disabled by the VA. And I think most of the medical evidence to support that came throughout the last, you know, [*6–10*] years or something like that... And I was getting, you know, maybe a 20% or 10% disability rate at the time. So I guess if I need to pick something that the VA could have done, it would have been recognize those injuries as being more I guess, disabling than they acknowledged at the time.”*

They also suggested that receiving training on the steps involved in applying for the VA disability benefits would be helpful, as people expressed knowing it existed but not how one qualifies or applies.

Participant 18: *“Of course, we knew about it. You know, we all know about the disability thing. None of us know how to go about it. When we first start, very daunting, you know. And like when I got out, it was very, you know, I’m thinking VA disability, you gotta have Agent Orange, been shot blown up and stuff like that. You know, but those are all things that my wife and myself have had to find out our own through reaching out.”*

#### Theme 3: Enhance access to mental health care.

Participants suggested that being offered treatment options for their mental health and substance use problems could have helped prevent their social struggles and homelessness. Some noted that the Army’s culture normalized, rather than helped mitigate, drinking and having support to cut down after leaving active duty would have been useful.

Participant 27: *“Ensuring that mental health treatment was followed through. I mean they knew I was screwed up when they cut me loose. And they just expected me to find my way in there or I’m not sure what the deal was. They promote drinking. They, you know, this whole rough and rowdy lifestyle of all the boys going out and this that and the other and then they try to pretend like we’re disease when we get home... it’s madness, the whole thing.”*

Participant 6 noted that being offered rehab sooner while in service would have been helpful, as he failed a drug test but was not offered treatment. Participants also stated that though they had connections to VA medical centers, the drive was long. For instance, one participant described a 45-minute drive and no car to get there, as well as a 4.5 month wait for an intake appointment. Participant 16 suggested offering appointments by phone or online, or an email or alert system sent that said “*Are you dealing with this right now? Click here.*” to offer more streamlined help.

## Discussion

This study revealed three key, modifiable pathways to veteran homelessness: financial challenges while reintegrating from military to civilian life, planning challenges around this transition, and interpersonal disruptions upon returning to civilian life. These findings are consistent with what has been reported in the largely quantitative literature on this topic [5], though this study is novel in adding an in-depth qualitative examination of these factors, including perspectives on prevention, in a national sample of veterans. In terms of prevention, veterans felt that enrolling veterans in services prior to separation, decreasing barriers to receiving VA disability benefits, and enhancing access to mental health care could have helped prevent their homelessness; these factors have not been clearly reported by veterans in prior qualitative literature. Each of the three main pathways into homelessness identified is discussed further below.

Regarding financial challenges, the finding that the major challenge is not just obtaining employment, per se, but obtaining employment that provides sufficient money for housing, merits attention. Multiple participants needed to work more than one job to save enough to obtain housing. Prior qualitative work has found that veterans connected loss of employment or unemployment with homelessness [6,7], but difficulty finding employment that pays sufficiently for housing appears to be a more recent finding. Thus, it seems that increasingly, many available forms of work do not allow veterans to earn enough to secure housing at the current level of wages [14]. Though this challenge is true in the general population as well, it becomes especially difficult for veterans. A finding consistent with prior literature is that veterans in this study noted military training is often highly focused on warfighting skills, which does not leave a clear pathway to adapt these skills for the civilian workforce, especially in those who are young and without employment prior to enlisting [15]. Therefore, the suggestion by participants to connect to sources of employment and civilian jobs skills training prior to separation as a prevention strategy seems worthwhile. One example of this is the DoD SkillBridge program, an opportunity for service members to engage in industry training, apprenticeships, and internships during the last 180 days of service [16]. Given that this program has been in existence since 2011 but was not mentioned by participants, this may be an example of the need for veterans to be better informed and connected to resources that already exist.

Other financial challenges related to VA disability, such as a lengthy waiting process to receive disability benefits and perception of a high barrier to qualifying for disability leading to homelessness, were notable findings. Decreasing barriers to receiving VA disability was noted to be a means to prevent homelessness among this population. Barriers to receiving VA disability identified in prior literature include disparities and regional variation not explained by clinical severity [17], perceptions of malingering [18], and complexity and misunderstanding of the process [19]. Given that VA service-connected disability benefits are not guaranteed for all veterans nor instantaneous, it seems important to temper veterans’ expectations and encourage them to find other sources of income while a claim is being processed. Furthermore, it is notable that in October 2017, a revamping of the Benefits Delivery at Discharge program [20] allowed service members to submit a claim for service-connected disability from 90 to 180 days, not 60 days, before discharge, which may have reduced the number of people without disability determinations for long periods after discharge. While VA reports indicate this has improved timely access to disability benefits, peer-reviewed evaluation is needed on the impact of this change. It is also important to recognize that many veterans seeking or on VA service-connected disability can and do continue to work [21]. More research is therefore needed to understand what veterans are doing while they wait for claims decisions, and how their risk for homelessness may be mitigated during that time and in the event the claims decision is not in the veteran’s favor.

Regarding challenges with planning for their transition, participants’ perspectives that the information they received upon separating from the Army was not adequate despite the existence of transition programs is consistent with prior literature. Even though TAP has undergone a series of revisions including significant updates in 2019 [22], it has been suggested that the program continues to lack attention to key areas of functioning such as adjusting to new work or educational settings, financial management issues, meeting family transition needs, or finding housing [23]. One personal challenge raised in this study was difficulty paying attention in the transition classes, but it could also be that how and when important material is presented needs further examination. Similarly, our findings confirm it is not the lack of existence of transition programs that are the problem, but lack of knowledge of how to benefit from them. This is consistent with a 2019 Government Accountability Office report which found 45 programs across 11 agencies that help servicemembers, veterans, and families find jobs [24], but that veterans had difficulty discerning which programs are relevant and whether they qualify [25]. The suggestion by multiple participants to have proactive outreach and include a liaison that can help veterans navigate programs and continue to follow people in civilian life is therefore worthwhile. Participants also attributed personal challenges with money management and not wanting to ask for help, perhaps related to the military’s focus on self-reliance, as leading to homelessness. These factors make proactive outreach even more important. Prior studies have suggested that veteran access to case management supports is a protective factor against homelessness [26]. For instance, the VA’s Liaison program, which started in 2003, may serve as an important resource that veterans need to be better informed about, as it consists of nurses and social workers who coordinate the transfer of healthcare from the DoD to VA nationwide as servicemembers leave the military [27].

Regarding challenges related to relationships, our findings about the loss of a primary relationship upon reintegration, particularly through divorce, are consistent with prior literature, but specific circumstances in our study were different. For example, Metraux et al. found that job loss and financial hardship precipitated the breakup of long-term relationships, whereas our study found that the loss of relationship also precipitated financial hardships [6]. Metraux et al also found that transitioning to civilian life made recovery from substances easier [6], whereas in our study multiple people stated that the heavy drinking culture in the Army made it difficult to stop using once coming back to the civilian world, leading to relationship termination and loss of housing. Participants suggested ensuring faster access to mental health care as a means to prevent homelessness resulting from mental health issues. In particular, the suggestion of telehealth to offer greater access holds promise based on recent studies indicating high uptake of telehealth in homeless-experienced veterans [28,29]. Of note, the VA MISSION Act of 2018 expanded options for veterans to access private healthcare facilities paid for by the VA when the VA cannot provide timely or specialized treatment within a reasonable distance [30]. However, studies have since shown ongoing frustrations with the administrative aspects of accessing care including billing and prior authorizations [31]. Given that a suggestion for prevention was enhancing access to mental health care, further evaluation is important to determine how to decrease mental health care barriers among veterans.

### Limitations

This study has several limitations. First, while we addressed a gap in the literature by focusing on the role of the transition period in risk of experiencing homelessness after separating from the military, we did not explicitly explore veterans’ perceptions about early life experiences or military factors leading to their homelessness. Second, though the longitudinal nature of the STARRS-LS data is a strength in giving a diversity of perspectives over time, its drawback is that the separation dates of veterans spanned a large range between 2011–2022 and the year that veterans left the Army was redacted due to confidentiality protocols, thus it is hard to precisely ascertain what changes to Army services were in place for each transitioning soldier. Third, since interviews required using zoom, it is possible that there were veterans with limitations in technology access or proficiency who were not represented. Finally, gender/sex was not commonly brought up in interviews, thus further exploration of how these as well as other demographic factors such as race/ethnicity affected pathways into homelessness should be a focus of future work.

## Conclusion

This study provides novel qualitative findings about modifiable pathways to homelessness among transitioning veterans. Veteran perspectives on prevention offer directions to mitigate the risk of homelessness among those who serve our country and facilitate their transition to a stable and successful civilian life.
